# 3D chemical imaging in the laboratory by hyperspectral X-ray computed tomography

**DOI:** 10.1038/srep15979

**Published:** 2015-10-30

**Authors:** C. K. Egan, S. D. M. Jacques, M. D. Wilson, M. C. Veale, P. Seller, A. M. Beale, R. A. D. Pattrick, P. J. Withers, R. J. Cernik

**Affiliations:** 1 School of Materials, University of Manchester, Manchester, UK; 2 UK Catalysis Hub, Rutherford Appleton Laboratory, Research Complex at Harwell, Didcot, OX11 0FA, UK; 3 Science and Technology Facilities Council, Rutherford Appleton Laboratory, Harwell, Oxfordshire, UK; 4 Department of Chemistry, University College London, 20 Gordon Street, London, WC1H 0AJ, UK; 5 School of Earth, Atmospheric and Environmental Sciences, University of Manchester, Manchester, UK

## Abstract

We report the development of laboratory based hyperspectral X-ray computed tomography which allows the internal elemental chemistry of an object to be reconstructed and visualised in three dimensions. The method employs a spectroscopic X-ray imaging detector with sufficient energy resolution to distinguish individual elemental absorption edges. Elemental distributions can then be made by K-edge subtraction, or alternatively by voxel-wise spectral fitting to give relative atomic concentrations. We demonstrate its application to two material systems: studying the distribution of catalyst material on porous substrates for industrial scale chemical processing; and mapping of minerals and inclusion phases inside a mineralised ore sample. The method makes use of a standard laboratory X-ray source with measurement times similar to that required for conventional computed tomography.

The penetrative nature of X-rays is exploited in X-ray computed tomography (XCT) to reveal the internal three-dimensional (3D) structure of an object^1^. XCT finds multiple applications across a range of scientific disciplines, including: medical imaging^2^, security scanning^3^, industrial inspection and metrology^4^, materials science^5^, geoscience^6^ and archeology^7^, amongst others. The method consists of recording X-ray absorption images (known as radiographs or projections) of an object at multiple rotation angles. Contrast in each radiograph is provided by the differential absorption of the X-rays which is directly related to a materials local density. At a synchrotron x-ray source the image is often collected using monochromatic illumination, whereas for laboratory sources white beam illumination is normally used. Either way, the detector normally collects the transmitted photons with no discrimination in terms of photon energy. A mathematical algorithm is then applied to the data to reconstruct the 3D distribution of the X-ray attenuation (sample density). The gray level histogram is then used to segment the 3D image into phases or features which can then be quantified^5^. Most XCT systems operate in the hard X-ray range (approximately 10–100 keV) primarily using the bremsstrahlung and characteristic radiation resultant from high energy electrons impacting onto a metal target. In this energy range, for the majority of atomic elements in the periodic table, the main contribution to X-ray attenuation is the photoelectric effect, the magnitude of which is approximately proportional to the 4th power of the atomic number (Z) and inversely proportional to the 3rd power of the X-ray energy (E)^8^. Additionally, the atomic photoelectric absorption cross-section shows rapid changes at energies corresponding to core-electron states, the K, L, M-edges. Because these characteristic step-changes in absorption occur at well defined X-ray energies, it is feasible to use these edges to identify individual chemical elements inside the object, as well as reconstruct the sample’s absorption contrast. All that is required to obtain this additional chemical sensitivity is the ability to precisely tune the X-ray photon energy to an absorption edge. Using the extremely bright X-rays generated at synchrotron radiation facilities, it is possible to use highly-selective optics (e.g. monochromators and focusing mirrors) that can produce radiation with a very narrow bandwidth (down to the 0.01% level) which can be tuned to specific absorption edges. 3D chemical imaging can then be performed by twice repeating the same tomographic scan at two different X-ray energies, one slightly above and one slightly below the desired absorption edge^9^,^10^. A basic subtraction of these datasets reveals the 3D distribution of the chemical element corresponding to the absorption edge. This technique has been exploited in areas such as electrochemistry^11^ and biomedical research^12^. An alternative method for 3D chemical imaging is to use the X-ray fluorescence (XRF) signal emitted after absorption. In this case a small, intense beam of X-rays from a synchrotron source is focussed down to a point and scanned across the object recording weak XRF signals at each point. This is then repeated at distinct small rotation steps of the object, and a reconstruction algorithm is used to build a 3D map of the atomic elements in the sample^10^,^13^. This approach has additional sensitivity but is much slower due the beam scanning process. It has been exploited in areas such as environmental chemistry^14^, biological science^15^, earth and planetary science^16^, and materials science^10^.

Unfortunately, both of these methods require the use of a synchrotron radiation facility, which have a major limitation from a throughput and access point of view inhibiting wide-scale adoption. Laboratory-based XCT instruments do not have the same limitations in terms of access. Due to the low intensity radiation emitted from a laboratory X-ray tube it is very time-consuming to undertake XRF imaging, while the low flux means CT is normally undertaken using a white beam ruling out imaging either side of an absorption edge. Over recent years, however, a range of pixelated spectroscopic X-ray detectors have been actively researched and developed which have the ability to measure X-ray energy or count the number of X-ray photons in specific energy bands with positional sensitivity^17^,^18^,^19^,^20^,^21^,^22^. These devices can be roughly grouped into two categories. The first category comprises the so-called single photon counting or multispectral detectors which count every single photon separately by analysing the electrical pulse generated when the photon deposits its energy in a detector pixel. This pulse is compared to that from one or more references energies enabling images to be grouped into a series of spectral bands. These devices generally have a coarse energy resolution (typically 5–10 keV) and can normally have up to 5–10 energy bins^23^. An advantage of multispectral detectors is that they can operate at relatively high count rates. A second category comprises the so-called hyperspectral detectors which measure the energy deposited in each pixel during a certain exposure time (or frame) and consequently calculate the associated photon energy of that pixel in that frame. This repeated over many thousands of frames where events are binned into a spectrum per pixel. The advantage of these types of detectors is that they can achieve a finer energy resolution (typically less than 1 keV) and can image over hundreds of spectral bands.^24^,^25^,^26^. However, due to the detector readout and associated processing, the measurement speed and maximum count rate is limited.

Multispectral detectors have been used in several laboratory-based X-ray imaging studies illustrating the advantages of spectral imaging for differentiating materials^22^,^27^,^28^. An obvious application is in medical imaging and tomography for obtaining enhanced contrast between similar density tissues or in the identification of tracer markers like gadolinium or iodine^21^,^29^,^30^. For such applications, whereby a great deal of information is already known about the sample composition and/or tracer markers have been used which have widely spaced K-edges, a multispectral detector is more than adequate. However, for materials science applications where the sample composition is often unknown beforehand or the sample contains multiple atomic elements that have closely spaced K-edges, we require an X-ray detector with a finer energy resolution—a hyperspectral detector.

Here, we demonstrate that it is possible to undertake chemically sensitive laboratory computed tomography by installing a hyperspectral pixelated detector (with a very high energy-resolution) inside a commercially available laboratory-based XCT system using the broadband bremsstrahlung radiation emitted from a microfocus X-ray tube. In this way we can identify multiple chemical elements simultaneously in 3D without prior knowledge of sample composition and with micron scale resolution. As we will demonstrate, the method has applications to a wide range of scientific disciplines, particularly materials science. Additionally, the ability to perform this kind of analysis in the laboratory (as opposed to a synchrotron) massively opens up the scope of the technique. In order to investigate the feasibility of the method and its practical utility we consider two examples: the first relevant to the chemical and catalytic sciences and the second relevant to geochemistry and environmental sciences. We start by describing the principle of the method as shown in Fig. 1; it is fundamentally analogous to that of regular laboratory-based XCT. X-rays generated from a microfocus X-ray tube illuminate a sample object and the projected radiograph is recorded on an area detector. The geometric magnification generated by moving the sample closer to the X-ray source facilitates micrometer scale spatial resolution. In this case however, we replace the conventional area detector with a HEXITEC hyperspectral detector which can record the absorption spectrum in each pixel with an energy resolution of less than 1 keV over the range of 10–100 keV^17^ (for more information on the detector, please see the methods section). By taking the natural logarithm of the normalised intensity in every spectral band we obtain a linear measurement of the absorption cross-section as a function of X-ray energy. A step-change in the attenuation spectrum indicates the position of an absorption-edge, which can be used to identify and map chemical elements in the sample. By rotating the sample and recording energy-sensitive projections it is possible to reconstruct the 3D volume of the sample object, but crucially each voxel now contains an absorption spectrum, as opposed to a single grayscale value, creating a 4D dataset (3 spatial dimensions, 1 spectral dimension). The position of an absorption edge in each reconstructed voxel spectrum is used as a characteristic marker, enabling chemical elements to be mapped in 3D.

Our first example application concerns the 3D distribution of catalytic metals supported on porous substrates, known as catalyst bodies. These are mm-sized gas porous support units (typically cylinders or spheres) onto which metals and metal oxides are deposited for the purpose of performing large-scale industrial catalysis. They are loaded in their thousands into industrial scale reactor units forming packed catalyst beds. The functioning of the bed and hence the efficiency of the entire chemical process is dependent on the performance of the individual catalytic body and as such it is important to understand their preparation and chemical form in 3D. The catalyst body provides a fairly inert and mechanically robust porous microstructure onto which a catalyst metal can be thinly dispersed and fixed in position by chemical bonding to the substrate. By choice of the preparation route, the catalyst can be directed to a desired distribution to maximize efficiency, both in terms of producing the desired industrial product and also minimising the amount of catalyst used. For example, expensive precious metals such as Pd are widely employed for both hydrogenation and oxidation reactions on an industrial scale, so any saving in terms of reactor efficiency or a reduction in the quantity of the catalyst metal required in the reaction, will correspond to large financial savings^31^,^32^.

A cylinder shaped γ-Al_2_O_3_ pellet with approximate diameter of 3 mm and height 3 mm was loaded with Pd metal *ex-situ* and scanned by hyperspectral XCT (see methods section for experimental details). Figure 2 shows the results from this scan—the data was reconstructed with a 53 μm voxel size. Figure 2a shows a typical absorption spectrum for a single voxel taken from the 3D reconstructed data volume. The first thing to note is the y-axis scale and units, here we use voxel optical density (VOD): 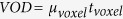 where μ_voxel_ is the voxel linear attenuation coefficient and *t*_*voxel*_ is the voxel size, as such *VOD* is a dimensionless quantity. We use this construct, as opposed to the linear attenuation coefficient, because generally the absolute value of the measured attenuation coefficient cannot be relied upon. This is due to various non-linear effects in the data acquisition and handling process, such as inter-pixel charge sharing, inter-frame charge sharing and reconstruction artefacts (see methods section and Supplementary Materials). Relative changes in the absorption spectrum can be reliably reconstructed, for example the position of an absorption edge. Unsurprisingly, in this data we observe a large step in the spectrum at 24.3 keV, corresponding to the Pd K-edge (24.350 keV). The detector has sufficient energy resolution in order to discount the possibility of confusion with adjacent elements in the periodic table (for example confusion of Pd with Rh at 23.220 keV or Ag at 25.514 keV—see Supplementary Materials). Using this data, it is possible to perform semi-quantitative analysis by spectral profile fitting (Fig 2b). We took a region of interest along the spectral domain around the K-edge and performed least squares fitting of linear functions to the data before and after the edge (red lines). By evaluating both linear functions at the K-edge position (24.350 keV, vertical dotted line) and subtracting the result, we get a measure of the magnitude of the absorption step, Δμ_0_. By doing this analysis we have removed the contribution of the voxel size on the optical density, but more importantly we have decoupled the individual contributions to the total absorption from the γ-Al_2_O_3_ and Pd metal, giving us a measure of the absorption cross section from Pd alone. This is important since it cannot be assumed that the density of the alumina is homogeneous throughout the sample. If we scanned this sample using a regular (non-spectroscopic) XCT system, it might be assumed that the observed high density regions correspond to that of Pd. However, this assumes that the alumina pellet has a homogeneous density, and realistically, for a systematic study the pellet should be pre-scanned before metal deposition so a subtraction can be performed. This is not practically possible in many circumstances, and assumes that the alumina pellet is not damaged during processing. This *in-situ* capabilty really highlights the power of hyperspectral XCT. Additionally, Δμ_0_ is directly proportional to the concentration of the metal species. By performing voxel-by-voxel fitting in this manner, we can build a 3D dataset of Δμ_0_ which can be processed and visualised in 3D. In order to obtain full quantitative information (e.g. absolute concentrations in weight percentage) it would be necessary to run a series of standard samples, having different sizes and concentrations. This is a non-trivial and complex task with many parameters to understand and there is a clear need for future work in this area. Figure 2c,d show vertical and horizontal slices through the Δμ_0_ 3D dataset, respectively. From these slices we can see that Pd has penetrated into almost all of the γ-Al2O3 pellet at varying concentrations. More importantly, regions of high concentrations can be seen, in particular around the periphery of the pellet, but also at certain ‘hotspots’ and at the surfaces of some of the large voids. Figure 2e shows a 3D exterior visualisation of the Pd distribution, where we can see the Pd appears to agglomerate around the circumference, but to a lesser extent on the top and bottom faces of the pellet; in good agreement with previous studies on these samples^33^.

In our second case study, we investigate the 3D distribution of mineral phases and inclusions in a mineralised ore sample. The sample is taken from a hydrothermal vein from the Leopard Mine, Silobela, Zimbabwe, part of the ~2.6Ga, Greenstone-hosted auriferous mineralisation in the Zimbabwe craton^34^. The sample comprises pyrite (FeS_2_), quartz (SiO_2_), gold (Au) and minor amounts of galena (PbS), chalcopyrite (CuFeS_2_) and bornite (Cu_5_FeS_4_). Optical 2D examination of polished sections of this sample reveals quartz and euhedral pyrite (mm to cm sized) to have precipitated first in the vein and been subsequently brecciated with the angular pyrite fragments re-cemented by quartz derived from later fluids. Gold precipitation was coeval with this second quartz episode and occurs as irregular masses (mostly <100 μm) and in fractures replacing the pyrite. Gold also occurs as small masses in the quartz and at quartz-pyrite interfaces. The other sulfides forms as irregular (<50 μm) inclusions in the pyrite. The pyrite-gold association is common in many exploited ore deposits due to gold absorption to the negatively charged pyrite surface precipitated from hydrosulphidogold(I) complexes or by the reduction of Au-chloride species^35^,^36^; the presence of arsenic in the pyrite can concentrate the gold.

Understanding the distribution of gold in pyritic and other mineralogically complex ores is important in the development of extraction processing pathways. Economic gold deposits often involve Au in very low concentrations (<10 gm/t) and the gold is as discrete grains. 3D chemical imaging has the potential to provide definitive information on the size, distribution and mineralogical association that will control liberation during processing. As gold mining has increasingly used heap leaching^37^ to extract gold, chemical XCT imaging during processing could have an important role in explaining extraction rates and allowing pathway tuning. Furthermore, the search and exploitation of new mineral resources to replace exhausted deposits and satisfy increasing demand will employ chemical or bio-leaching^38^ of large volumes of low grade ore and 3D chemical imaging has the potential to play a significant role in ensuring efficient exploitation.

A 20 mm diameter core sample was scanned using the spectroscopic XCT system—the data was reconstructed with a 65 μm voxel size. Figure 3a shows a grayscale tomographic slice through the sample produced by integrating over the full spectral range. Different mineral phases are observed by their variation in gray level contrast. Brecciation of the pyrite can be seen, particularly highlighted towards the bottom of the image. Figure 3b shows two voxel spectra from two separate inclusions. The first spectrum shows a step change in attenuation corresponding to the Au K-edge (80.725 keV) whilst the second spectrum shows a Pb K-edge (88.005 keV). The measured position of these edges positively differentiates the gold and galena (PbS) mineral phases. In order to segment and separate these two phases we used a K-edge subtraction method. By extracting images at energies above and below the desired absorption edge and subtracted them, we produced Au only and Pb only 3D datasets. A vertical slice through the grayscale data is shown in Fig. 3d with the segmented Au and PbS phases—coloured blue and red respectively—overlaid. Both particles are embedded in pyrite. An important feature of this data is that due to inter-particle variations in the attenuation, it was not possible to accurately segment the Au and PbS phases using grey level (energy indiscriminate) contrast. Some of the gold inclusions show a much higher density which can be easily segmented using a threshold, but many particles show either similar gray level contrast, or a much lower contrast to that of PbS. As such, on the basis of data produced by scanning this sample in a non-spectroscopic (standard) XCT system, it would not be possible to reliably differentiate or segment PbS from Au. Only the K-edge sensitivity of the spectroscopic XCT system can produce a positive identification and therefore successful elemental segmentation. Figure 3c shows voxel spectra from three other mineral phases in the sample: quartz, pyrite and chalcopyrite. These minerals show no observable absorption edges since they contain only low Z-atomic elements with edges below the sensitivity of the system. They can be easily segmented based upon their relative attenuation contrast. In this case however, we exploited the spectroscopic nature of the data and segmented based upon a least square fit of a linear function to the absorption spectra in the range 60–90 keV. The gradient of the linear function is used as a segmentation tool by thresholding. This approach gives better inter-phase distinguishability since it is less sensitive to image noise and was particularly useful in the quartz mineral due its low attenuation and high level of reconstruction artefacts. 3D visualisations of the distributions of each segmented mineral phase, including Au and Pb containing particles, are shown in Fig 3e. From this data we found that the Au inclusions are almost always embedded in pyrite, whereas Galena inclusions can be either embedded in pyrite or quartz. Many inclusions may be below the resolution of the system, either spatially—i.e. smaller than the voxel size of the scan (65 μm), or spectrally—i.e. with concentrations below the sensitivity limit of the system. With regards spatial resolution, chemical XCT can ultimately produce voxel sizes on a par with current microtomography scanners (<5 μm) and is only really limited by the number of pixels on the detector. For this sample with a 20 mm diameter, it could be possible to achieve resolutions of around 10–15 μm, given a large enough field-of-view. With a smaller sized sample it could be possible to achieve <5 μm resolution. With regards chemical sensitivity, we have yet to perform a systematic study, however previous experience suggests a limit of detection of around 1–2%wt per voxel.

The above two case studies have highlighted the capabilities of laboratory-based 3D chemical imaging, however there are of course limitations. Most prevalent of these is sample self-absorption. Let’s take a theoretical example to demonstrate this: Say we wanted to map the distribution of Mo in a sample of Ti 6246 alloy. This alloy is frequently used in applications where high strength and light weight are needed along with good corrosion resistance. Understanding the distribution of alloying elements in this material, particularly operating under real-world conditions (an attribute of non-destructive XCT) might be of interest^39^. The Mo K-edge is at 20.000 keV, so for absorption edge identification we require X-ray photons of this energy to be able to pass all the way through our sample. If the sample is too large or too dense, too few K-edge photons will make it to the detector and we will not be able to identify Mo in the sample. The attenuation length of 20.000 keV photons in Ti metal is about 140 μm. An approximate upper limit on the maximum feasible sample size is roughly twice the attenuation length (i.e. 13.5% transmission), so in this case about 300 μm. This represents a rather small sample, but not impractically so, for high resolution micro CT systems. A sample much larger than this and we will struggle to detect any characteristic photons and therefore be unable to identify this element. So in practice there are limitations on sample size, but also on sample density and as such the method is best suited to studying moderate-to-low density samples containing heavy elements (high atomic numbers—high energy K-edges). Having said that, it is feasibly possible to study lighter elements from the periodic table given the right experimental conditions, for example, the first row transition metals (e.g. Ti, V, Cr, Mn, Fe, Co, Ni, etc.). These elements have K-edges that are below the sensitivity of the detector used in this paper (<10 keV), however using an appropriate low energy spectroscopic detector (e.g. a silicon pixelated detector with a very high energy resolution^26^,^40^) it may be possible to map the distribution of such elements, given a suitably small and low density sample. A previous study demonstrated the ability to identify K-edges below 10 keV using such a detector^40^. In the same work, a CT scan was performed on a mouse artery injected with a gold-containing contrast agent (looking for the gold L-edge at approximately 13 keV). The authors managed to infer the presence of gold and produce a 3D render based on density contrast in energy subtracted images. However they failed to make a positive identification due to a poor signal-to-noise ratio in their data. They also failed to identify the presence of Zn, Ca and Cu in their sample which they had previously identified by X-ray fluorescence. These shortcomings may be more down to current detector technology (in particular the count rate limitations of silicon based detectors) as opposed to a fundamental inadequacy of absorption edge tomography at lower X-ray energies. As detector technology improves, sensitivity to these lighter elements will no doubt improve, as will spatial resolution.

In summary, we have developed a laboratory-based XCT system that has the ability to produce 3D images with chemical sensitivity and micrometer scale resolution. By exchanging the regular area detector on an XCT scanner with a pixelated spectroscopic X-ray detector (with a very fine energy resolution <1 keV) we can obtain hyperspectral 3D datasets with voxels containing absorption spectra. Step-changes in the absorption spectra signify the position of absorption-edges which are used as a fingerprinting tool to identify chemical elements inside each voxel. A particular attribute of this method is that it can be very simply retrofitted in to most currently available XCT systems, simply by adding in a spectroscopic detector, in turn enabling wide scale adoption and expanding the available range of materials characterisation techniques. This can be exploited further by utilising the non-destructive nature of X-ray tomography to study 3D chemical processes occurring inside materials and structures which are functioning under real-world conditions (e.g. temperature, pressure, corrosive environments etc.). We expect this method will find applications to a wide range of scientific disciplines covering materials science (e.g. elemental mapping in metal alloys), planetary, earth and environmental sciences (e.g. siting and association of toxic elements in highly complex materials such as soils), chemistry and catalysis (e.g. *in-situ* monitoring of catalytic and synthesis reactors), and archaeology (e.g. non destructive evaluation of metal antiquities).

## Methods

### X-ray detector

A HEXITEC spectroscopic detector was installed in a Nikon XTH 225 system. The HEXITEC detector consists of a 1 mm thick CdTe single crystal detector (20 × 20 mm^2^) bump-bonded to a large area ASIC packaged with a high performance data acquisition system. The detector has 80 × 80 pixels on a 250 μm pitch with an energy resolution of 800 eV at 59.5 keV and 1.5 keV at 141 keV^17^. During operation each photon event has its energy, pixel position and the frame in which it occurs recorded. Events are processed and histogrammed according to measured energy into 0.25 keV wide bins. We typical use between 400–800 bins, depending on the maximum X-ray energy. Normally, during this process, a correction is employed to deal with photons that may have shared its energy between two or more pixels which appear to be measured as multiple lower energy photon measurements on neighbouring pixels. When the flux is sufficiently low it is possible to identify these shared events and reconstruct the correct photon energy in the correct location. However due to a high flux of radiation and therefore a high percentage occupancy of events per frame (making it very difficult to identify shared events), we did not employ a charge sharing correction strategy in this case. Previous studies have shown that this does not significantly impact on the measured position of an absorption edge^24^,^41^. An inter pixel energy calibration was performed using a correlative optimised warping algorithm using data from a flat-field fluorescence image off a series of metals^42^.

### Data acquisition

The Nikon XTH 225 system was used with a microfocus (spot size <5 μm) reflection Cu target X-ray tube which was operated at a beam current of 15 μA. For the catalyst pellet sample, we recorded 60 projections covering 360 °, with an exposure time of 150 seconds per projection (total scan time of 2.5 hrs–60 kV tube voltage). For the geological core sample, we scanned the detector along 5 separate positions in the horizontal direction to increase the field of view. Each sub-projection was recorded for an exposure time of 45 seconds (225 seconds for a full projection) and we performed 120 projections covering 360 ° (total scan time 7.5 hrs–160 kV tube voltage). All tomographic reconstructions were performed using a GPU accelerated simultaneous iterative reconstruction technique operating in series on each energy channel, producing a 4D dataset (3 spatial dimensions, 1 spectral dimension). A wavelet based Fourier filter was applied to the sinogram before reconstruction to suppress ring artefacts^43^.

### Catalyst sample preparation

A batch of five Pd catalyst bodies were prepared by pore-volume impregnation with Pd(NH3)4Cl2.H2O pre-cursor (110 ml of a 4 wt% solution). These were shaken for 5 min, dried (120 °C for 120 min) and then calcined (ramp 50–500 °C at 4.5 °Cmin^−1^; hold 60 min at 500 °C; cool down at 4.5 °Cmin^−1^). A single catalyst body was selected from this batch and scanned using the hyperspectral XCT system.

## Additional Information

**How to cite this article**: Egan, C. K. *et al.* 3D chemical imaging in the laboratory by hyperspectral X-ray computed tomography. *Sci. Rep.* **5**, 15979; doi: 10.1038/srep15979 (2015).

## Supplementary Material

Supplementary Materials

## Figures and Tables

**Figure 1:**
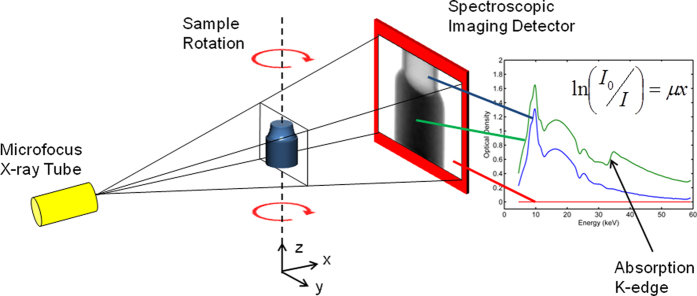
Hyperspectral XCT: A regular area detector in a commercially available microtomography scanner is replaced with a spectroscopic imaging detector with a high energy resolution. The absorbed X-ray spectrum in each pixel can now be measured. A geometric magnification can be defined by moving the sample closer to the X-ray source enabling micrometer scale resolution. By recording projections at different angles of sample rotation, a 3D dataset of the sample object can be mathematically reconstructed. Each voxel now contains an absorption spectrum as opposed to a grayscale value. Step-changes in the absorption spectrum signify the position of absorption edges, which can be used to map the distribution of chemical elements inside the object with micron scale resolution.

**Figure 2:**
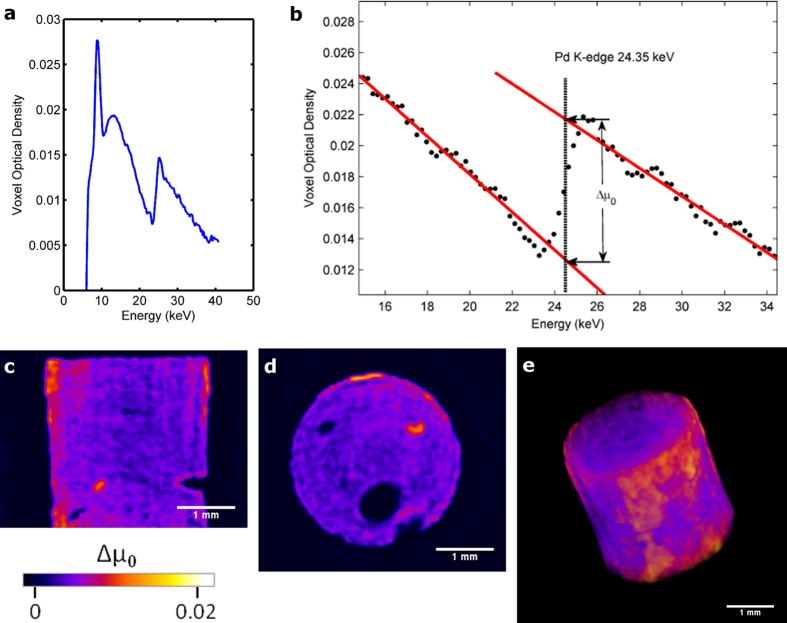
Pd metal distribution on a porous alumina catalyst body. (**a**) Typical voxel spectrum from the sample showing a distinct step-change in attenuation at 24.3 keV, corresponding to the Pd K-edge. (**b**) Spectral fitting to the absorption spectrum. Two linear functions are least squares fitted above and below the edge. These are evaluated at the known edge position and subtracted to obtain Δμ_0_. By repeating this process for every **voxel** spectrum, the relative Pd metal concentration can be visualised in 3D. (**c**) and (**d**) Vertical and horizontal orthoslices of Δμ_0_. (**e**) 3D exterior visualisation of Δμ_0_.

**Figure 3:**
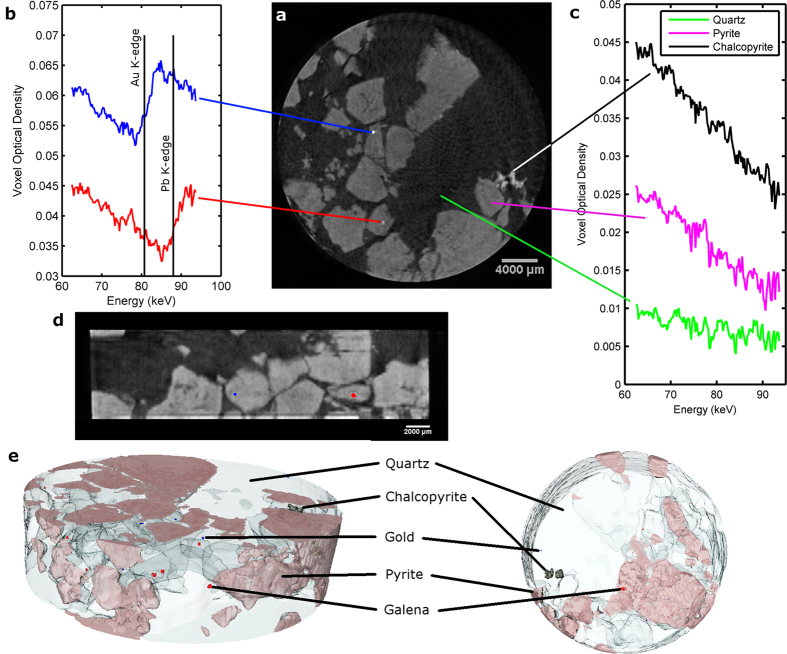
3D distribution of mineral phases in a mineralised ore sample from a gold-rich hydrothermal vein. (**a**) Grayscale tomographic slice through the sample created by integrating over the full spectral range. (**b**) Voxel spectra showing **Au** and Pb K-edges. (**c**) Voxel spectra from quartz, pyrite and chalcopyrite minerals. (**d**) Vertical slice through the sample with Au (blue) and Pb (red) containing voxels segmented and coloured. (**e**) 3D visualisations of the distribution of mineral phases in the sample.
