# Supplementary material for: 3D chemical imaging in the laboratory by hyperspectral X-ray computed tomography
Source: Sci Rep. 2015 Oct 30;5:15979. doi: 10.1038/srep15979 (PMC4626840; doi:10.1038/srep15979)
Supplement: Supplementary Materials [file srep15979-s1.pdf]

Supplementary Information:

3D chemical imaging in the laboratory by hyperspectral X-ray computed tomography

C. K. Egan[1], S. D. M. Jacques[1,2], M. D.Wilson[3], M. C. Veale[3], P. Seller[3], A. M. Beale[2,4], R. A. D. Pattrick[5], P. J. Withers[1], R. J. Cernik[1]

[1] School of Materials, University of Manchester, Manchester, UK

[2] UK Catalysis Hub, Rutherford Appleton Laboratory, Research Complex at Harwell, Didcot, OX11 0FA, UK

[3] Science and Technology Facilities Council, Rutherford Appleton Laboratory, Harwell, Oxfordshire, UK

[4] Department of Chemistry, University College London, 20 Gordon Street, London, WC1H 0AJ, UK

[5] School of Earth, Atmospheric and Environmental Sciences, University of Manchester, Manchester, UK

Edge position accuracy

For the catalyst body sample described in the main text, we wanted to be confident that the edge position that we can see is definitely from that of Pd, rather than that of any other element, for example, the adjacent elements in the periodic table (Rh or Ag). Figure S1 shows a zoomed in region around the measured K-edge in the catalyst body sample. Overlaid are the theoretical K-edge positions of three consecutive atomic elements in the periodic table (Rhodium, Palladium and Silver). Given the high energy resolution of the HEXITEC detector, the measured step-edge position verifiably corresponds to that of Pd.

In order to establish the edge resolution across the full energy range of the detector we have recorded edge profiles for a number of different metal foils using typical exposure times used in a single projection. The exact edge positions can be extracted by least squares fitting an arctangent function to a pixel spectrum, using a quadratic function to account for the background signal:

$$y = ax^2 + bx + c + d\tan^{-1} e(x - x_0)$$

By repeating this for multiple spectra, we can extract mean values and standard deviations typically using >100 pixels for each test sample. Figure S2 shows an example fit to an absorption edge spectrum for an indium metal foil (600 µm thick; X-ray tube 70 kV, 0.15 µA, 60 seconds exposure, detector 0.25 keV bin width). Even with this rather noisy data, the absorption edge can be clearly seen and succesfully fitted using the arctangent function. The edge position can be accurately found. Figure S3 shows a plot of various measured K-edge positions from a series of thin metal foils, as a function of atomic number, Z. Overlaid in red is a plot of the theoretical K-edge positions for those atomic elements. Error bars are plotted as 5x standard deviations to make them more visible. Typically, using this method the position of an edge can be measured to within 150 eV (1 standard deviation) - which is approximataly 5 times better than the energy resolution of the detector (800 eV).

Absolute measurement of attenuation

Given the ability to precisely count and measure the energy of photons it should be possible to obtain an accurate absolute value of the attenuation coefficient which can be used to map back specific materials in the sample (i.e use the absolute value of the absorption cross section rather than the K-edge to obtain chemical snesitivity). However, due to various non-linear effects in the X-ray detection process, this is not practically the possible. The most prevalent of these limiting effects is inter-pixel charge sharing which gives erroneous values for the number and energy of the photons 'seen' by the detector. It is feasible to correct for this in the detection process, however in this case due to the (relatively) high flux seen by the detector it cannot be done. This is due to the high percentage occupancy in individual frames (typically about 40-50% events/pixel/frame) a charge sharing correction cannot be applied since it is impossible to distuinguish shared events from coincidence events. Another factor is inter-frame sharing, where events in the same pixel are shared between consecutive frames. Further factors include, detector quantum efficiency, charge trapping/loss and tomographic reconstruction artefacts. In summary, the absolute value of the linear attenuation cannot be accurately measured largely because we cannot employ a charge sharing correction algorithm, but other factors also play their part. Fortunately, this does not impact on the ability to 'see' absorption edges, as can be seen by the myriad of data presented in this paper.

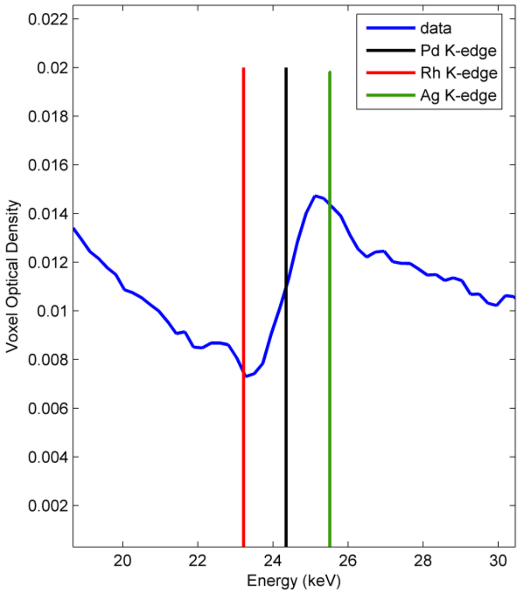

Figure S1: Rh, Pd and Ag K-edge positions overlaid onto experimental data of the Pd absorption edge from the catalyst pellet sample in the main text. The edge position matches well to that of Pd.

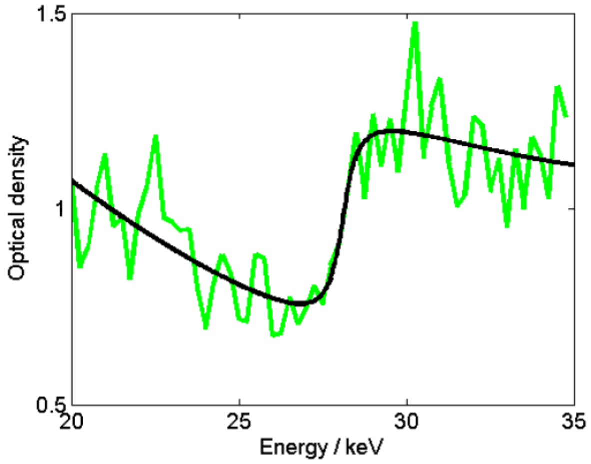

Figure S2: Example least squares fit (black line) of an arctangent function to experimental data (green line) of the indium K- edge from a metal foil.

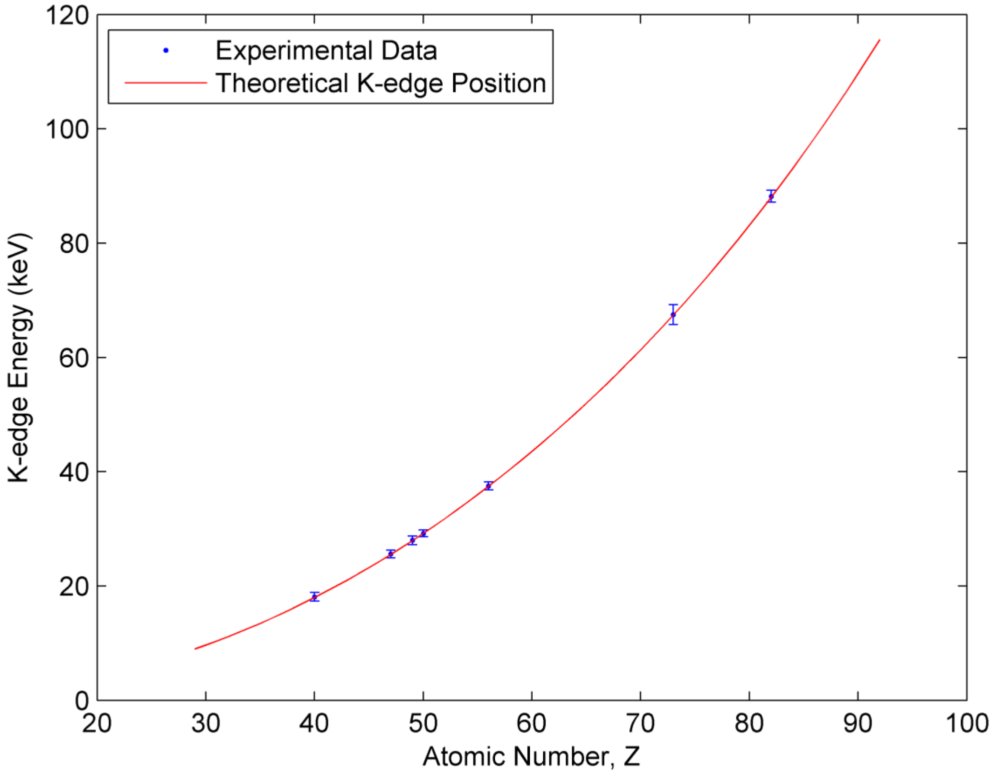

Figure S3: Measured K-edge positions from a series of metal foils found by fitting an arctangent function to experimental data. Error bars are shown as 5x standard deviations to make them more visible. The red line shows the theoretical K-edge position of each element as a function of atomic number, Z.

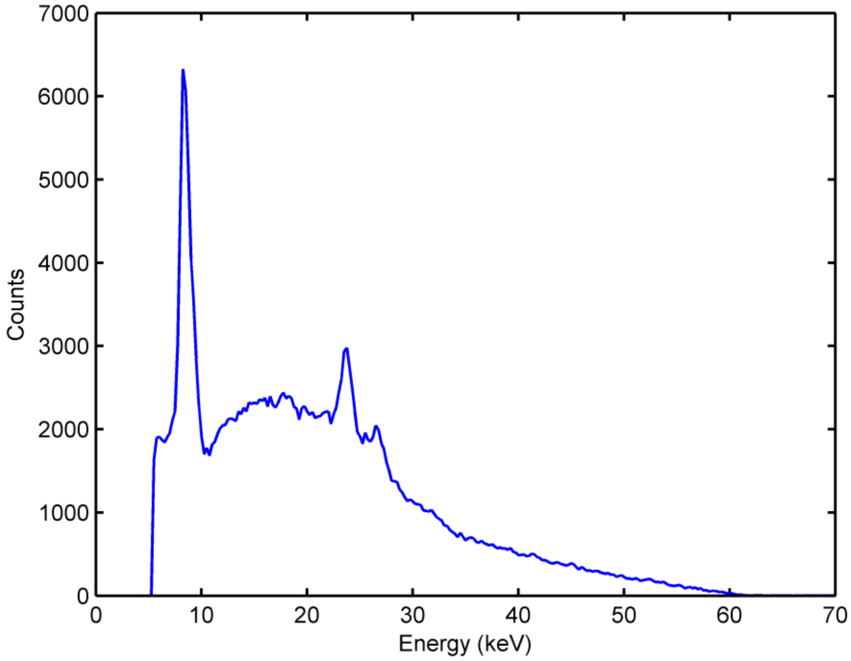

Figure S4: Typical incident source spectrum as measured by one pixel of the detector. 60 kV, 15 µA, Cu target, 0.1 mm Al filter, 150 second exposure.
